# The State of Lunacy in Ireland

**Published:** 1849-07-01

**Authors:** 


ART. III.—*Lunatic Asylums, Ireland. Report on the District, Local, and Private Lunatic Asylums in Ireland,* 1848, *with Appendices, presented to both Houses of Parliament, by command of her Majesty.* Dublin: Thorn. 1849.

THE state of lunacy in Ireland has been repeatedly a matter of parliamentary inquiry, and now, when distress, famine, pestilence, and voluntary self-expatriation are spreading desolation through that unhappy land, the subject appears to us invested with a deeper and more solemn interest than heretofore.

The want of proper accommodation for the insane and the lunatic poor of Ireland was long ago a national grievance. Prievous to the year 1810, no provision had been made by the legislature for this class of sufferers beyond a few cells in the workhouses, and county prisons, and St. Patrick's Hospital, which was opened upon the 19th of September, 1757, and is now capable of receiving 150 patients. In the year 1810, a grant was made by parliament for establishing the Richmond Lunatic Asylum in Dublin, for the accommodation of 200 patients, [55 Geo. III. c. 107,] which was opened in 1815; but the sphere of its operation, extending through the counties of Dublin, Meath, Wicklow, and Louth, embraces a population of 180,984 persons, and it is shown by the tables before us that its institution is wholly inadequate to meet the wants of so extensive a district.

In 1817, a committee of the House of Commons reported that the only effectual mode of relief would be the establishment of district asylums, *eleven* of which are now open; but although they have been considerably enlarged and extended, the provision they afford is wholly inadequate to meet the wants of the lunatic poor in each district. The result is that patients are still consigned to gaols and cells in workhouses, "where these poor creatures are often obliged thus to remain until vacancies occur in the asylums proper for their reception."—(*Report,* p. 5.) The impropriety, or rather the absolute inhumanity, of such a system is obvious. In these temporary places of refuge, proper care and medical treatment in the early stages of the disease are impracticable; the disorder, consequently, becomes aggravated and confirmed, and to this cause may now be ascribed the fact that the district lunatic asylums are now encumbered with hopeless and incurable cases. The Report states—

"The evils arising from the practice of committing lunatics to gaols, and the want generally of adequate asylum relief, increased to such an extent as to be made the subject of a parliamentary inquiry, in the year 1843, when a select committee of the House of Lords was appointed to examine into the state of the Irish asylums, with the view of remedying these defects.

"The result of their labours will be seen in their report of the evidence taken before them, and their views, after a careful examination of that evidence, are generally embraced in the following resolutions—viz.,

"'The committee are desirous of impressing on the House, as the result of their inquiries, the following propositions, on which they have formed the strongest opinion—

1. The necessity of discontinuing, as soon as practicable, the committals of lunatics to gaols and Bridewells.
2. The necessity of amending the Act of the 1 Vict. c. 27, which appears, on the authority of the Lord Chancellor of Ireland, to have led to the most serious abuse.
3. The inexpediency of appropriating the union workhouses as places either for the custody or the treatment of the insane, for both which purposes they appear wholly unsuited.
4. The necessity of providing one central establishment for criminal lunatics, under the immediate control and direction of the government of Ireland, to be supported from the same funds and under the system adopted in respect to criminal lunatics in England.
5. The necessity of increasing the accommodation for pauper lunatics in Ireland, and of providing for the cases of epilepsy, idiotcy, and chronic disease, by an increased number of the district asylums, by an enlargement of those asylums, or by the erection of several establishments specially appropriated for these classes of patients.'

"The valuable suggestions thus thrown out by the committee were taken up by the Irish government the following year, and a correspondence opened with the grand juries of each county, and their opinion requested on the elegibility and centrality of certain sites proposed for the new asylums, which the inspectors-general of prisons were instructed to inspect, and report on for the information of the lord-lieutenant.

"The limited powers of the Executive under the existing enactments, interposed an obstacle to any further progress beyond mere preliminary arrangements, with the exception of some additions to the asylums, called for by the pressure of demands for admission in some of the districts. But it is now gratifying to state, that the evil has been at length remedied, by the Act of last session,

8 & 9 Vict., c. 107, which removes the legal restriction that has hitherto limited the accommodation of each asylum to 150 inmates, and thus prevented their enlargement to any sufficient extent. It also provides for the erection and establishment of a central asylum for criminal lunatics, which will relieve the existing asylums from this class of patients."—(*Report*, p. 6.)

Notwithstanding these laudable intentions promulgated in 1846, we perceive by the present Report, that on the 31st December, 1848, there were, out of an aggregate of 5678 patients—338 confined in gaols, and 1940 in union workhouses. Furthermore, from another table, exhibiting the number of committals of lunatics to gaols, and their discharges, we observe that during the years 1847 and 1848, there were as many as 1041 lunatics committed to gaol, without being convicted of any offence beyond their being unhappily so afflicted. This is not all. The inspectors report that they "have also reason to believe that family disputes, particularly where land and other property is in question, have occasionally led to the confinement of individuals, [under the Act, 1 Vict. cap. 27,] some provocation to an act of violence being given in order to bring the parties within the more effectual cognizance of the law."—(*Report*, p. 8.) To provide against this evil, the inspectors require a copy of the certificate of admission of every dangerous and criminal lunatic; but even this appears to us insufficient. The practice of committing lunatics to gaol ought to be abolished; nay, although a majority of them be minor culprits, their offences are probably the result only of their insanity. We regret, therefore, that such a system as this should still exist in Ireland. The provision for the insane in workhouses, although only temporary, is equally objectionable; indeed we cannot conceive a more deplorable picture than the inspectors themselves give us in the following statement:—

"The wards allocated to lunatics in the workhouses of this country, however ill-constructed and ill-adapted to the purpose, may be looked upon as so many subsidiary depots affording a temporary shelter to the insane, in the absence of suitable accommodation. Originally intended for idiots, as their name implies, *these wards become from time to time receptacles for patients labouring under every type of mental disease. On our visitations, we have occasionally found in them cases of the most acute mania, requiring a care and vigilance that could not be expected from a class of attendants taken out of the general mass of paupers, frequently aged and infirm themselves, and whose services are for the most part unrequited.* If admission to the district asylum cannot be procured for individuals thus acutely affected, no alternative is left, but to await a vacancy, unless in the interim, as usually happens, they are committed to the nearest prison, as dangerous, or perhaps as criminal lunatics. The great majority, however, of the insane in unions, may be classified as idiotic, epileptic, and demented, amounting in all to 1943. There being no law nor fixed regulation to confine persons labouring under these different grades of mental aberration to a continued residence in unions, when once admitted, their numbers in them are necessarily subject to much fluctuation. *Of* 130 *poorhouses,* 124 *at present contain a certain proportion of lunatics, ranging from four to over sixty. In some unions, during the two past years, such was the pressure from without, and so great the necessity of hospital accommodation, that the idiot divisions, when not fully occupied, were done away with altogether, and made available for general purposes, their former inmates being diffused through the paupers at large.* We are aware that the Commissioners for the Relief of the Poor in Ireland, as well as the local authorities, are alike sensible of the great inconvenience caused by the residence of the insane, no matter of what class, in workhouses, where, generally speaking, *cooped up in gloomy cells, without any provision for exercise, comfort, or employment, any attempt at classification, or moral treatment on their behalf, is quite impracticable.*"

Such a description as this—the sane mingling with the insane—the poor idiot with the pauper—cooped up in gloomy cells, and wholly unprovided for with either moral or medical treatment, carries us back to the remoter and darker age when insanity was little understood, and the unhappy victim was left, as in Hogarth's picture, to pine away in straw and misery. Such is at this moment, it would seem, the state of lunacy in Ireland.

Another point for regret which is suggested by the Report before us is, the want of any definite statistical account; inasmuch as we find by the Report of 1846, that the number of pauper and other lunatics in Ireland were disposed of as follows :—

| IN ASYLUMS, OR OTHERWISE ACCOMMODATED. | IDIOTS. | | | EPILEPTICS. | | | MANIA. CURABLE. | | | MANIA. INCURABLE. | | | TOTAL. | | |
|---|---|---|---|---|---|---|---|---|---|---|---|---|---|---|---|
| | M. | F. | Total. | M. | F. | Total. | M. | F. | Total. | M. | F. | Total. | M. | F. | M. & F. |
| District Lunatic Asylum . . . . . | 91 | 87 | 178 | 119 | 55 | 174 | 355 | 404 | 759 | 746 | 698 | 1,444 | 1,311 | 1,244 | 2,555 |
| Local ditto . . . . . . . . . . | 72 | 112 | 184 | 21 | 35 | 56 | 20 | 15 | 35 | 116 | 171 | 287 | 229 | 333 | 562 |
| Gaols . . . . . . . . . . . . | ... | ... | ... | ... | ... | ... | ... | ... | ... | ... | ... | ... | 177 | 113 | 290 |
| Poor Houses . . . . . . . . . | 440 | 533 | 973 | 212 | 377 | 589 | 5 | 47 | 52 | 97 | 210 | 307 | 754 | 1,167 | 1,921 |
| Total in District Asylums, Gaols, and Poor Houses . . . . | ... | ... | ... | ... | ... | ... | ... | ... | ... | ... | ... | ... | 2,471 | 2,857 | 5,328 |
| Wandering cases of Idiots and Simpletons, not in any Asylum, (from Returns furnished in 1844) . . . | ... | ... | ... | ... | ... | ... | ... | ... | ... | ... | ... | ... | 3,992 | 2,225 | 6,217 |
| Gross Total of Pauper Lunatics . | ... | ... | ... | ... | ... | ... | ... | ... | ... | ... | ... | ... | 6,463 | 5,082 | 11,545 |
| In Private Asylums . . . . . . . | 6 | 9 | 15 | 6 | 1 | 7 | 51 | 34 | 85 | 92 | 52 | 144 | 155 | 96 | 251 |
| Lunatics under care of Court of Chancery, not in any Asylum . . . . | ... | ... | ... | ... | ... | ... | ... | ... | ... | ... | ... | ... | 47 | 29 | 76 |
| Total in private Asylums, and under protection of Court of Chancery | ... | ... | ... | ... | ... | ... | ... | ... | ... | ... | ... | ... | 202 | 125 | 327 |
| Total in Public Asylums, Gaols, and Wandering cases . . . . | ... | ... | ... | ... | ... | ... | ... | ... | ... | ... | ... | ... | 6,463 | 5,082 | 11,545 |
| GRAND TOTAL . . . . | ... | ... | ... | ... | ... | ... | ... | ... | ... | ... | ... | ... | 6,665 | 5,207 | 11,872 |

Hence at this period, on the 1st of January, 1846, there were 11,872 lunatics in Ireland; and yet in the report now published, we find that the aggregate accommodation which will be provided by the new and existing asylums will only afford reception for 4500 inmates. It is stated, and the fact is melancholy, that the famine and pestilence of 1847, which pressed so heavily on the pauper population, visited with peculiar severity the insane. The inspectors report, that "as a class no portion of the community suffered more than the destitute, whether labouring simply under aberration of mind or a total deprivation of reason. At a period of aggravated misery, when individual life depended upon individual exertions, or personal appeals to charity, it cannot be a matter of surprise that the unprotected lunatic or idiot, in whom physical debility is for the most part combined with mental imbecility, should be amongst the first to suffer. Destitution itself was no unfrequent cause of madness; independent of direct cases, which fell under our own cognizance, we have unerring testimony to the fact, in the palpable increase of applications for admission to asylums in a diminished population; and in many instances, it would seem that the insanity arising from starvation was a mere prelude to death, as the comforts and remedial treatment of our hospitals were found inoperative to restore either health or reason. To these causes, combined with an epidemic dysentery, would we attribute the unusual and extensive mortality which prevailed during 1847 and the commencement of 1848 amongst the insane. The deaths in district asylums alone amounted in 1847 to 422, and in gaols to 122, exceeding those of the preceding year by 224 and 47 respectively. In poor-houses, the disproportion of mortality between these two years was much greater, as might be expected, in consequence of the more rapid propagation of disease from their overcrowded state. In some of the southern and western unions, the inmates of the idiot wards were almost all carried off by fever or other complaints; and, as a case in point, we may adduce Bantry, where, in the course of a few months, out of thirteen of these afflicted beings we found but three remaining."—(*Report*, p. 6.)

Such a calamitous season vitiates, *quoad* that period, any ordinary statistical induction; but this is not all. In the table above given for the year 1846, there is a return of "wandering cases of idiots and simpletons, not in any asylum, from reports furnished in 1844, amounting to 6217;" and in the present Report, which appears to us very defective upon this point, we gather, that according to the returns furnished by the constabulary, the number of wandering lunatics and imbeciles amounts to 6000, although the inspectors add, that in some remote districts "they may be considered almost to be extinct"—as if, indeed, they were Saurian reptiles, deserving no better fate. But take the number, as given by the constabulary, at 6000, we shall then have in Ireland :—

| | | |
|---|---|---|
| Lunatics in district asylums | . . . . . . | 2,603 |
| " local asylums | . . . . . . | 365 |
| " private asylums | . . . . . . | 432 |
| " gaols | . . . . . . . . . . | 338 |
| " union workhouses | . . . . . | 1,940 |
| | Total . . | 5,678 |
| Wandering lunatics and imbeciles | . . . . | 6,000 |
| | Total . . | 11,678 |

And yet the accommodation contemplated in the improvement and enlargement of existing asylums will not exceed admission for more than 4500. This is truly lamentable!

Again, the inspectors in the Report before us attest their belief that these wandering lunatics and idiots, by intermarriage, actually propagate the disease. "We may here," they observe, "express our belief, as the result of observation and inquiry, that insanity in all its varied forms can be occasionally traced, even through remote degrees of relationship, from its original propagation by wandering idiots and imbeciles, and that the practice of intermarriage, which prevails in some districts, from a desire of the parties to perpetuate the tenure of land or other property amongst their immediate kindred, is not an unfrequent cause of lunacy; in evidence of which, we might instance the fact of ten of the same family being committed, in the course of a few years, as dangerous lunatics alone to the gaol of Monaghan."

With an increasing population, we are well aware that insanity will also be on the increase; but the ratio of insanity to the population in England, Scotland, Wales, and Ireland is different. The inspectors in this Report calculate, that in England the number of persons labouring under mental disorder is to the general mass as 1 to 870; in Scotland and Wales, 1 to 740; while in Ireland, it is 1 to 900. We, however, repeat, that the data for any accurate induction is obviously so defective, that we cannot come to any satisfactory result. In Ireland, as in England, there is a large class of lunatics unprovided for—persons who are not exactly paupers, but enabled to pay a certain sum, although small, for their board and residence. "I am most anxious," said Dr. Conolly, in his evidence before the committee of the House of Lords, in 1843, "to see such institutions in this country. There is not a week passes without my having the most affecting accounts of distress brought upon families in middle life by the insanity of some relative—the head, perhaps, of the family—or a son, or a daughter, and there is no place to send them to. We want an institution that should receive them for a small sum, within their ability to pay—say 20*l.* or 30*l.* a year, other patients being received at higher rates, but all being accommodated alike."

The same want appears to exist in Ireland. "We regret," say the inspectors, "that as yet no provision exists for the insane, who not being paupers, are legally inadmissible into our public institutions, and who at the same time are unable to meet the charges generally made in private licensed houses. The individuals in question principally belong to the farming classes, and as they are heavily assessed for the erection and support of our district asylums, we think, under certain restrictions, they have an equitable claim to enjoy the benefit of such public establishments as paying patients, and at an average cost of maintenance.

"The only mixed institutions into which the insane are received at a moderate rate are St. Patrick's Hospital and the Retreat; the accommodation in them is, however, far too restricted for general purposes, and they are consequently more appropriated for lunatics from the better classes of life, and who may have fallen into straitened circumstances. Of the manner in which they are conducted we take the present opportunity to express our approval; and it is a circumstance of pride to us to state that the asylum originally founded by the celebrated Dean Swift, when the treatment of lunacy was little studied, and humanity towards the insane still less practised, was and has ever continued, from the liberality and benevolence of its governors, as well as the high professional character of its attendants, a credit to the country."

Besides the district and local asylums, there are also private establishments, principally in the vicinity of Dublin and Cork, for the reception of patients in the higher ranks of life. These, with one or two exceptions, are under the management of members of the medical profession. With these private asylums the inspectors state they have every reason to be satisfied, and they trace a continued improvement in such houses, and the social and domestic condition of their inmates.

"We have also observed that a strong disposition exists on the part of proprietors to make up for primitive defects by structural improvements, which are being effected in many licensed houses, and will, we anticipate, be extended to all; and notwithstanding the disadvantage arising from their not having been originally intended as abodes for the insane, it is gratifying to report that an effective classification is preserved, and that in some instances completely detached buildings have been licensed and appropriated for the exclusive occupation of the respective sexes—an arrangement much to be approved of.

"Due provision has also been made for religious service, and the patients have full opportunity of conforming to their respective persuasions: as the amusements and occupations must principally depend on the character of the disease, and the progress towards recovery; for those who are not allowed beyond the precincts of the asylum, various social recreations are provided, such as music, reading, drawing, &c.; whilst convalescents in a more advanced stage are permitted to drive, walk out, and occasionally go to concerts, and other places of amusement, under proper surveillance; there is every reason to believe that this indulgence induces a feeling of contentment, and tends towards the eventual recovery of the patients.

"On our various visitations to licensed houses, we have made their domestic arrangements particular subjects of inquiry, and amongst other matters, we had occasion to direct that a greater variety of diet should be supplied in some, with a more suitable attendance on the patients. We also found it our duty to communicate directly with the immediate relatives of parties respecting clothing and other requisites, where satisfactory evidence was adduced to show that the remonstrance of the superintendent or proprietor had failed in obtaining the necessary supply; and we required, in more than one instance, the deficiency to be provided for at the asylum. Our interference may have thus occasionally extended beyond the special injunctions imposed on us by law; but feeling it incumbent, as a matter of duty, we have not failed to exercise the discretionary powers vested in us by the Act of Parliament to the fullest extent, wherever our official interposition could in any way advance the social condition, or promote the comforts of the lunatic."—(*Report*, p. 12.)

The inspectors, in their present Report, make several suggestions, which may be very advantageously adopted. In reference to the profits arising from the industrial employment of patients, they truly observe—

"That habits of industry are far from being incompatible with the loss of reason, or an original deficiency of intellect. Independent of farming operations and common household trades, many are employed in the manufacture of cloth, linen, lace, &c., the saving from which amounted in the last two years to 3629*l.* We would here take the opportunity to suggest, that a certain portion of the net profits might be both charitably and judiciously laid aside for the benefit of convalescents, particularly females, so as to secure them a temporary support after their discharge, and to obviate the danger of a relapse from penury and destitution."

They very properly advise that clergymen, whether Catholics or Protestants, should be attached to every asylum.

"There are at present but three district asylums which are not regularly attended by paid chaplains; and though visited from time to time by the parochial clergy, as such visits are gratuitous and uncertain, we have recommended to the boards the propriety of adopting a system which elsewhere has been found beneficial. In one of the establishments referred to, objections may arise, from the variety of religious persuasions in it; but as a large proportion of the inmates belong to the Established and Roman Catholic Churches, it is highly desirable to have chaplains respectively for them, with a Presbyterian clergyman for the others generally."

They also propose that the resident managers of all hospitals for the reception of the insane shall hereafter be professional men, which is clearly a proper suggestion; for whatever be the form of the malady, every patient ought to be at all times under medical surveillance.

The inspectors, in conclusion, comment on the defective state of the law of lunacy in Ireland, and repeat their recommendation in a former Report, that the several Acts now in operation be consolidated into a single statute.

"Our sphere of duty," they observe, "having become gradually extended much beyond its original limits, and finding our usefulness, on the one hand, daily impaired by the defective provisions of previous acts, and our exertions, on the other, continually retarded by the circuitous interposition of powers conferred under the still older enactments for public asylums, dating some of them more than twenty years back, we would desire to impress upon your lordships' attention that the exigencies of the Irish department require an enactment such as that already in force in England; we do not, therefore, hesitate to recommend a consolidation of the different lunacy Acts at present in force in Ireland, feeling convinced that the interests of this branch of the public service demand that a bill should be submitted to parliament during the coming session, which would embrace within the *compass of a single statute*, a combined system of laws to regulate both the public and private lunatic asylums of this country.

"We have thought it our duty to submit to your lordships' consideration this expression of our conviction, feeling assured that the existing English enactment, and the bill before parliament for Scotland, afford, with the experience of this department, a strong precedent, and sufficient materials from which to frame a similar measure, adapted to the character and wants of our Irish asylums; and when the occasion presents itself, we will endeavour, by our suggestions on the several points in detail, to show that a central authority, with an organization like that extended to England, and contemplated for Scotland, could be effectually applied in this country to carry out an improved system of superintendence and management, and in a manner calculated to secure an uniform and effective administration of our Irish institutions."

In bringing our analysis of the Report before us to a conclusion, we would observe that at a period of Irish history so fraught with domestic and political strife, setting at bold defiance the concentrated wisdom of the British legislature to find for the many evils which exist an efficient remedy, how is it possible for us to expect that the wants of the insane portion of the population can be sufficiently provided for in a manner at all commensurate with their unhappy and deplorable condition? The attention of government is, we are glad to hear, directed to these crying evils, and every disposition exists, on the part of the Irish authorities, to meet the exigencies of the case. We must not expect, for the present, great things; but we have no doubt that, ere long, a just and comprehensive enactment will receive the sanction of the Parliament, which will, as far as such enactments can do, grapple with this important subject.

